# Efficacy and Tolerability of a *Scutellaria lateriflora* L. and *Cistus* × *incanus* L.-Based Chewing Gum on the Symptoms of Gingivitis: A Monocentric, Randomized, Double-Blind, Placebo-Controlled Clinical Trial

**DOI:** 10.3390/nu16060862

**Published:** 2024-03-16

**Authors:** Alessandro Di Minno, Hammad Ullah, Lorenza Francesca De Lellis, Daniele Giuseppe Buccato, Alessandra Baldi, Paola Cuomo, Hesham R. El-Seedi, Shaden A. M. Khalifa, Xiang Xiao, Roberto Piccinocchi, Gaetano Piccinocchi, Roberto Sacchi, Maria Daglia

**Affiliations:** 1 Department of Pharmacy, University of Naples “Federico II”, Via D. Montesano 49, 80131 Naples, Italy; hammad.ullah@unina.it (H.U.); lo.delellis2@libero.it (L.F.D.L.); d.buccato@studenti.unina.it (D.G.B.); alessandra.baldi.alimenti@gmail.com (A.B.); 2 CEINGE-Biotecnologie Avanzate, Via Gaetano Salvatore 486, 80145 Naples, Italy; 3 Department of Agriculture, University of Naples “Federico II”, Via Università 100, 80055 Naples, Italy; paola.cuomo@unina.it; 4 Department of Chemistry, Faculty of Science, Islamic University of Madinah, Madinah 42351, Saudi Arabia; elseedi_99@yahoo.com; 5 Psychiatry and Psychology Department, Capio Saint Göran’s Hospital, Sankt Göransplan 1, 112 19 Stockholm, Sweden; shaden.khalifa@regionstockholm.se; 6 School of Food and Biological Engineering, Jiangsu University, Zhenjiang 212013, China; xiaoxiang1@aliyun.com; 7 Level 1 Medical Director Anaesthesia and Resuscitation Azienda Universitaria Ospedaliera Vanvitelli, Luigi Vanvitelli, Via Santa Maria di Costantinopoli, 80138 Naples, Italy; roberto.piccinocchi@policliniconapoli.it; 8 Comegen Azienda Universitaria Ospedaliera Vanvitelli, Società Cooperativa Sociale di Medici di Medicina Generale, Viale Maria Bakunin 41, 80125 Naples, Italy; gpiccino@tin.it; 9 Applied Statistic Unit, Department of Earth and Environmental Sciences, University of Pavia, Viale Taramelli 24, 27100 Pavia, Italy; roberto.sacchi@unipv.it; 10 International Research Center for Food Nutrition and Safety, Jiangsu University, Zhenjiang 212013, China

**Keywords:** *Scutellaria lateriflora* L., *Cistus × incanus* L., gingivitis, chewing gum, randomized clinical trial

## Abstract

Preclinical studies have shown that the combination of *Cistus* × *incanus* L. and *Scutellaria lateriflora* L. extracts exerts beneficial effects on oral health against gingivitis. Thus, this study aimed to assess the tolerability of a chewing gum and its efficacy on gingivitis in a double-blind, placebo-controlled clinical trial. Enrolled subjects (*n* = 60, 18–70 years) were randomized to receive two chewing gums or a placebo daily for 3 months. At baseline (t0) and monthly (t1, t2, and t3) timepoints, the Quantitative Gingival Bleeding Index (QGBI), the Modified Gingival Index (MGI), and the Oral Health 15 items (OH-15)] were employed to assess potential improvements in gingivitis. Pain was self-quantified via the Visual Analogue Scale (VAS), and the Clinical Global Impression Scale for Severity of illness (CGI-S) helped in evaluating the oral general conditions. This study is listed on the ISRCTN registry. At t3, the QGBI, MGI, OH-15, VAS, and CGI-S values decreased in the treated but not in the placebo group (β = 0.6 ± 0.1, t_176_ = 3.680, *p* < 0.001; β = 0.87 ± 0.21, t_115_ = 4.263, *p* < 0.001; β = 5.3 ± 2.5, t_172_ = 2.086, *p* = 0.038; β = 3.16 ± 0.51, t_88_ = 6.253, *p* < 0.001; and β = 1.09 ± 0.32, t_83_ = 3.419, *p* < 0.001, respectively). A significant improvement in gingival health occurred after a 3-month intervention with the chewing gums containing *S. lateriflora* and *C. incanus* extracts.

## 1. Introduction

Gingivitis is a common, mild form of periodontal disease (gum disease) causing tenderness, redness, and swelling of the gum around the teeth [1]. If not promptly handled, gingivitis spreads to the underlying tissue and bone (periodontitis), ultimately causing tooth loss [2]. Poor oral hygiene is the most common cause of gingivitis, which leads to the formation of dental plaque, and inflammation of the surrounding gum tissues. Plaque [3] can stabilize below the gum line and turn into tartar, which makes plaque removal challenging [4]. Tartar also provides a protective shield for bacteria [5]. The microorganisms most associated with gingivitis include *Streptococcus*, Fusobacterium, *Actinomyces*, Veillonella and *Treponema*, Bacteroides, *Capnocytophaga*, and *Eikenella* species [6]. The longer plaque and tartar stay on the teeth, the more gum swelling and easy bleeding occur [7].

For the removal of dental plaque and tartar from the teeth, antibiotic therapy (such as tetracycline, metronidazole, doxycycline, amoxicillin, azithromycin, clindamycin, chlorhexidine, and spiramycin) and the daily use of antiseptic mouthwash or toothpastes targeting the main causative agents of periodontal disease are the most common treatments against gingivitis and the other periodontal diseases [2,8,9]. The most used chemical plaque control agents are chlorhexidine, cetylpyridinium chloride, sodium fluoride, stannous fluoride, stannous chloride, zinc oxide, and zinc chloride, which have been proven effective in preventing plaque accumulation and in the prevention and treatment of periodontal diseases [9]. In recent years, a growing interest in the use of plant extracts in toothpastes and mouthwashes, to avoid the increasing emergence of multidrug-resistant pathogens and to meet market demand for natural, well-tolerated, and effective plaque control agents, has led to an increase in the use of natural phytochemicals derived from plants in oral hygiene products. In particular, plant extracts with antimicrobial and anti-inflammatory activities present possible alternatives to chemical plaque control agents. Here, the combination of two chemically characterized commercial extracts obtained from the aerial parts of *Scutellaria lateriflora* L. and *Cistus × incanus* L. was tested. The main bioactive compounds present in the extracts are represented by flavonoids (e.g., quercetin, kaempferol, myricetin) [4]. These entities are commonly used as food supplement ingredients and have previously been studied for their antimicrobial activity against *Porphyromonas gingivalis*, one of the major pathogens responsible for severe and chronic periodontitis. The protective effects manifested in a dose-dependent manner against cellular invasion by *P. gingivalis* and presented antibiofilm activity [4]. Moreover, these extracts were found to contain very complex mixtures of polyphenols and were found to be quite stable following in vitro simulated oral digestion.

Considering (1) that bacteria cannot be completely removed from the surface of the teeth by mechanical procedures; (2) the long-term use of antibiotics and antiseptic agents carries the risk of promoting the development of resistance and side effects; and (3) the promising in vitro results obtained through a preclinical study on the chemically characterized *S. lateriflora* and *C. incanus* extracts [4], these commercial plant extracts were used to prepare chewing gums to ensure prolonged, close contact of the extracts with the masticatory system and potentially combat the symptoms of gingivitis by improving oral health. Thus, the tolerability and the efficacy of a *S. lateriflora* and *C. incanus*-based chewing gum was investigated, employing a monocentric, placebo-controlled, randomized, parallel-group, double-blind trial.

## 2. Materials and Methods

### 2.1. Food Supplement and Placebo

In this trial, a food supplement based on a chewing gum containing the hydroalcoholic extracts of *S. lateriflora* and *C. incanus* (aerial part), commercially available as a functional raw material (Planoràl^®^ EPO S.R.L., Milan, Italy), was investigated. Details of the extracts, their chemical characterization, in vitro bioaccessibility, and antimicrobial activities are thoroughly reported in a previously published paper [4]. The gum otherwise consisted of sweeteners (i.e., isomalt, sorbitol, xylitol, maltitol syrup, sucralose, acesulfame k); a gum base; flavorings (menthol, eucalyptus); spiruline; safflower; coating agents (carnauba wax); antioxidants (tocopherol-rich extract, E306); a stabilizer (glycerol); and a thickener (Arabic gum). Two chewing gums provide a daily dose of 160 mg of the Planoràl^®^. Aside from the hydroalcoholic extracts of *S. lateriflora* and *C. incanus* (aerial part), all other ingredients present in Planoràl^®^ were present in the placebo preparation. To guarantee double blinding, the placebo was unrecognizable from the chewing gum containing the *S. lateriflora* and *C. incanus* extracts in packaging, color, shape, weight, and flavor. Both the verum and placebo, produced within European specifications for contaminants and microbiologic limits by INDACO S.p.A., Caivano (Naples, Italy), were provided by EPO S.R.L. as promoters of the clinical trial. The food supplement had been previously notified at the Italian Health Ministry (notification number: 153637).

### 2.2. Volunteers

Sixty subjects of either sex, between 18 and 70 years of age and able to understand and sign on informed consent, were included in the study. The participants were recruited following the inclusion criteria reported below by the general practitioner in the presence of a dentist who made the diagnosis and performed the test for the calculation of the Quantitative Gingival Bleeding Index (QGBI). The visits were carried out at the outpatient setting of the general practitioner group that operatively organized the clinical trial, whose protocol was approved by the ethics committee. No incentives were offered to the recruited participants. All participants had gingivitis in the absence of diagnosed periodontitis or tooth loss caused by periodontal disease. All had a cut-off value for the ‘Quantitative Gingival Bleeding Index (QGBI)’ score ≥ 1 [10] and cut-off value for the ‘Modified Gingival Index (MGI)’ score ≥ 1 [11]. All subjects scored ≥ 2 for the question “*Have you had any problems with bleeding gums?*” in the ‘Oral Health 15 items (OH-15)’ [12]. A minimum of 20 teeth present was the lowest limit for subjects over 60 years of age. *Exclusion criteria* from this trial comprised the following: pregnant or breastfeeding women; subjects suffering from overt periodontitis; those suffering from oral diseases (tongue cancer, glossitis, dry mouth, etc.), subjects who had undergone dental cleaning or scaling within 2 months of enrollment, subjects who had taken antibiotics within 2 months of enrollment, subjects with current use of, or that had used in the prior week, prescriptions of over-the-counter medications which may affect inflammation (i.e., NSAIDs), subjects with cognitive disorders that may have hindered the response to questionnaires; those with a history of allergy to the components contained in the study treatments; those with a history of addiction or abuse of medications, drugs, or alcohol; heavy smokers (≥10 cigarettes per day), and those with genetic–metabolic diseases, diabetes mellitus, rheumatological diseases, or neuropsychiatric or neurological diseases. Participants were informed about the components of the chewing gum and the possible beneficial and adverse effect of the treatment. Moreover, they received instructions about (1) chewing the gum for approximately 30 min (minimum 25 min and maximum 30 min) [13], (2) taking the treatments in the middle of the morning and middle of the afternoon (11 am and 5 pm, which allows the maintenance of the bioactive substances incorporated in the chewing gum in the oral cavity in the morning after breakfast and in the afternoon after lunch, and subsequently after brushing the teeth, for a good part of the daytime hours), (3) abstaining from consuming other food supplements, chewing gums, antibiotics, or FANS, for the entire duration of the study, (4) abstaining from consuming food or beverages, except water, 1 h before every study visit. The study design did not include measures of the patient’s compliance regarding these instructions. But compliance to the treatment consumption was measured during every study visit through the returning of empty/almost empty treatment packages.

### 2.3. Validated Questionnaires

The improvement of gingivitis symptoms was assessed by means of validated questionnaires.

The QGBI questionnaire [10] measures the extent of any blood coverage of the bristles of a toothbrush, following brushing and light pressure on the gums. This index is part of the so-called “bleeding on probing” tests [14,15], i.e., suitable indices to evaluate the degree of gingivitis: 0—no bleeding when brushing; absence of blood-stained bristles; 1—slight bleeding when brushing; presence of blood stains on the tips of the bristles; 2—moderate bleeding on brushing; about half the length of the bristles, from the tip down, stained with blood; and 3—severe bleeding on brushing; full length of all bristles including the head covered with blood.

The MGI questionnaire [11] is based on the doctor’s visual assessment of the degree of inflammation of the gum tissue: 0—normal gingiva; 1—mild inflammation—slight discoloration and slight edema; 2—moderate inflammation—redness, edema, and glazing; and 3—severe inflammation—marked redness and edema, and ulceration with a tendency to spontaneous bleeding.

The OH-15 [12], a 15-item self-assessment questionnaire, examines the symptoms of discomfort in the oral cavity in the 7 days prior to the completion of the study.

The indices from the medical evaluation of the health status of the gingival tissue and health, and of the validated self-assessment questionnaire, were also used during the screening visit to assess the eligibility of the subject (see inclusion and exclusion criteria section). The results (the sum) of the different items of these validated self-assessments are the primary response variable of the study.

The degree of pain was self-assessed (perceived gum pain) using the ‘Visual Analogue Scale (VAS)’ [16] at t0 (baseline), t1 (30 days of treatment), t2 (60 days of treatment), and t3 (90 days of treatment). The *VAS* ranges from one (no pain) to ten (the highest level of perceived pain). The effect of taking the supplement on the general conditions of oral health and gingivitis was assessed by the physician in parallel with subject’s self-assessment of the degree of pain through the questionnaire ‘Clinical Global Impression Scale for Severity of illness (CGI-S)’ at t0 (baseline), t1 (30 days of treatment), t2 (60 days of treatment), and t3 (90 days of treatment) [17].

### 2.4. Trial Design

This monocentric, randomized, placebo-controlled, parallel-group, double-blind clinical trial was performed by COMEGEN—Società Cooperativa Sociale (Naples, Italy). After being evaluated during the screening visit to establish whether the recruited subjects fulfilled the inclusion criteria, they were randomized with a simple randomization into two groups, using two chewing gums per day (approximately at 11.00 a.m. and 5.00 p.m.) containing *S. lateriflora* and *C. incanus* extracts (group 1) or two chewing gums per day of the placebo (group 2). At t0 (i.e., baseline), all enrolled subjects underwent an assessment of gingival health indices with the validated questionnaires administered by the investigating physician. The assessment of the gingival health indices, QGBI and MGI, and the completion of the OH-15 questionnaire and of the VAS, administered by the investigating physician, were also recorded at t1, t2, and t3 (i.e., at 30 days, at 60 days, and at 90 days after starting the study). The completion of the CGI-S questionnaire by the investigating physician was only carried out at t3. Clinical data were processed in accordance with current privacy regulations as required by Regulation (EU) 2016/679, and the clinical data of individual volunteers were processed anonymously. The trial was carried out according to the European Union’s Standards of Good Clinical Practice (Directive 2001/20/EEC) in accordance with the current Declaration of Helsinki, concerning medical research on humans (Helsinki 1964, amended by: Tokyo 1975, Venice 1983, Hong Kong 1989, Somerset West 1996, and Edinburgh), and the Guidelines for Good Clinical Practice (CPMP/ICH/135/95). All subjects to be enrolled in the study were informed about the aims of the study by means of an ad hoc understandable form approved by the ethics committee during the review of the protocol. All signed the informed consent. In the patient information, it was clarified that all the components of the chewing gum and placebo are permitted in food supplements and that the the research fulfilled institutional procedures and requirements. The protocol, letter of intent of volunteers, and synopsis of the study were approved by the local ethics committee of A.S.L. Napoli 1 CENTRO (protocol number 73, 14 February 2023). This study is listed on the ISRCTN registry (www.isrctn.com, accessed on 14 February 2023) under the ID number ISRCTN 16727861, https://doi.org/10.1186/ISRCTN16727861 (accessed on 15 January 2024) to make identification of the participants possible if needed (D.L. 52/2008).

#### 2.4.1. Outcomes of Study

The primary outcome was to measure gingival health indices and potential improvements in symptoms (compared to the placebo group) at the end of the three-month intervention with the food supplement. The improvement of gingivitis symptoms was assessed and measured by the investigating physician at t0, t1, t2, and t3 via the QGBI, MGI, and OH-15 questionnaires. The results (the sum) of the various items of these validated self-assessment questionnaires correspond to the primary response variable of the study.

With regard to secondary objectives, the perceived gum pain was self-assessed by the VAS at t0, t1, t2, and t3. The CGI-S questionnaire score at t0, t1, t2, and t3 was recorded by the investigating physician to evaluate the subject’s general oral condition.

#### 2.4.2. Data Collection

The data collection was carried out through a case reporting form (CRF) divided into two main sections. The first, completed at the time of the enrollment, was related to volunteers’ personal data, medical history, intake of concomitant medications, and treatment group. The second contained the results of the validated questionnaires i.e., QGBI indices, MGI, OH-15, VAS, and CGI-S.

### 2.5. Safety and Tolerability

No serious adverse events related to the intake of the food supplement were expected. However, the enrolled subjects were continuously monitored for the occurrence of any kind of adverse effects and, in the event that suspected adverse reactions occurred, these would be reported via the VigiErbe online phytovigilance system (www.vigierbe.it; accessed on 1 December 2023) [18], according to the provisions of the Istituto Superiore di Sanità. Subjects with allergies to any of the ingredients of the food supplement were categorically excluded from the study.

### 2.6. Statistical Analysis

The preliminary power analysis indicated a sample size of 56 subjects in total (28 subjects per group) to ensure a power of 95% with a significance of 0.05, assuming an average effect size (Cohen’s f = 0.20). Considering a drop-out of ≈10% of study subjects, the number of volunteers to be enrolled was increased appropriately: 60 subjects completed the two months of dietary supplement. The study design implies repeated measurements within each volunteer. The most suitable statistical analysis model for this study appeared to be a random intercept linear mixed model (LMM), where the dependent variable represented the scores of each questionnaire, in separate models, while the measurement (i.e., t0 to t3), group, age, and sex were the independent variables. The measurement × group interaction was added to the fixed effect to identify differences in the response to measurements of the two experimental groups. The individuality of each patient/volunteer was a random effect, controlled through within-subject repetition of the measurement. A preliminary analysis indicated that the OH-15 variable did not behave normally, even after log transformation, as this variable was used in the selection of volunteers, providing bias. Thus, the analysis was carried out on the changes in the index compared to the initial value (t0). Analyses were performed using the lme4 package [19] in R ver. 4.0.1 [20], and unless otherwise stated, data are reported as means ± standard deviations.

## 3. Results

**Figure 1:**
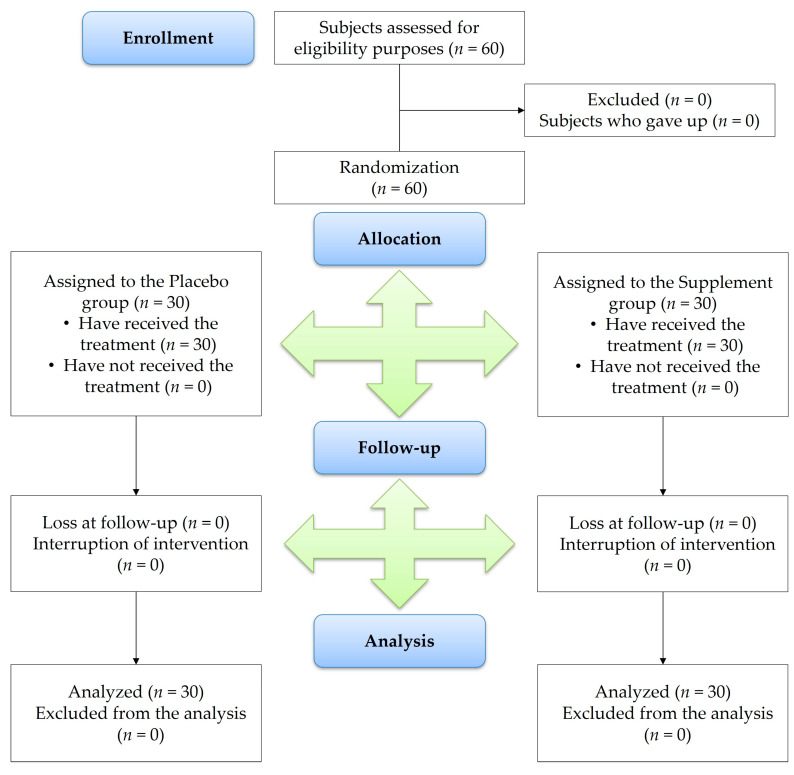
CONSORT PRO flow diagram.

The study flowchart, produced in accordance with CONSORT PRO reporting guidelines [21], is shown in Figure 1. The baseline characteristics of the subjects of each group are summarized in Table 1. Sixty subjects (*n* = 30, each group) with a total of 30 males (13 of whom were allocated to group 1) and 30 females (17 of whom were allocated to group 1) received their treatments daily for 3 months following enrolment. The participants presented similar sociodemographic characteristics and clinical data except from age. Despite a significant discrepancy in the age of the subjects assigned to the control and the placebo groups, the statistical analysis did not show a significant effect of age on the efficacy data collected. The food supplement was well tolerated, and no safety issues were determined during the trial. None of the enrolled subjects reported side effects or adverse reactions to the intervention, including any allergies.

**Figure 2:**
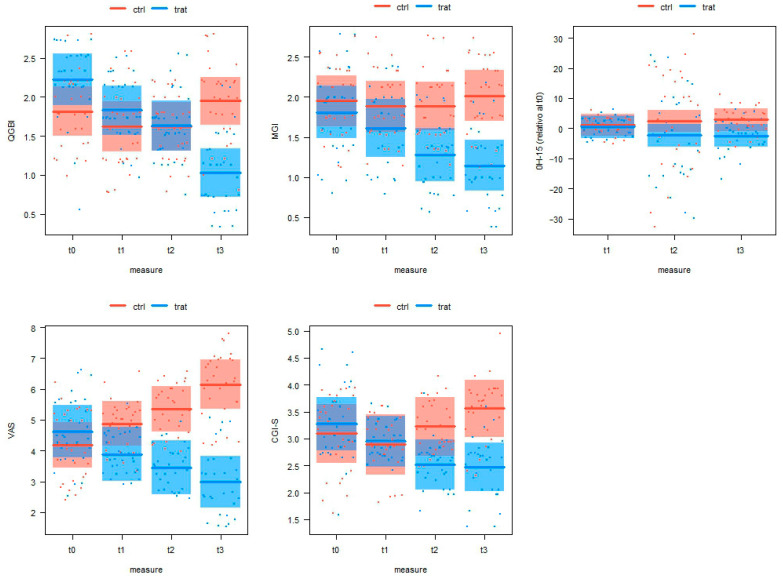
Changes in the primary and secondary outcome values (conditional effects redicted by the LMMs) in the placebo and treated experimental groups (placebo = red; food supplement = blue) at the four evaluation timepoints (t0: baseline; t1: 30 days of treatment; t2: 60 days of treatment; t3: 90 days of treatment). Horizontal lines are for means and boxes are for 95% confidence intervals (estimated by bootstrapping, *n* = 999).

Table 2 provides descriptive statistics for the values of the scores of the five outcomes at the four measurement points in the two experimental groups (group 1: placebo; group 2: food supplement). Table 3 and Table 4 and Figure 2 show the results of the LMM analysis. For the QGBI values, the LMM model shows highly significant effects (*p* < 0.001) for both the measurement and the measurement × group interaction in the absence of any significant effect due to sex and age (Table 3). The QGBI measurement values were lower in the placebo than in the treated group at t0 (β = 0.41 ± 0.21, t_115_ = 1.985, *p* = 0.049). The two groups did not differ from each other at t1 (β = 0.21 ± 0.21, t_115_ = 1.020, *p* = 0.31) and at t2 (β = 0.11 ± 0.21, t_115_ = 0.056, *p* = 0.95), while the QGBI value of the treated group was significantly lower than that of the placebo group at t3 (β = 0.92 ± 0.21, t_115_ = 4.446, *p* < 0.001). Thus, in the treated group, the value of the QGBI decreased during the trial (Figure 2). This decrease was statistically significant from t0 to t1 (β = 0.4 ± 0.1, t_174_ = 2.916, *p* = 0.004) and from t2 to t3 (β = 0.6 ± 0.1, t_176_ = 3.680, *p* < 0.001) but not from t1 to t2 (β = 0.2 ± 0.1, t_174_ = 1.458, *p* = 0.15). In contrast, a significant increase between t2 and t3 (β = 0.3 ± 0.1, t_174_ = 2.430, *p* = 0.16, Figure 2) was found between the QGBI values in the placebo group. 

The LMM model also identified significant effects for the MGI value measurements and their interaction in the absence of significant effects of sex and age (Table 3). Indeed, the MGI values did not differ between the groups, with t0 (β = 0.14 ± 0.21, t_115_ = 0.680, *p* = 0.50) and t1 (β = 0.27 ± 0.21, t_115_ = 1.332, *p* = 0.19). However, the MGI values of the treated group were significantly lower than those recorded for the placebo group at both t2 (β = 0.61 ± 0.21, t_115_ = 2.961, *p* = 0.003) and t3 measurement points (β = 0.87 ± 0.21, t_115_ = 4.263, *p* < 0.001). In the treated group, the MGI values decreased from t0 to t1 (β = 0.20 ± 0.13, t_174_ = 1.486, *p* = 0.14) and from t2 to t3 (0.13 ± 0.13, t174 = 0.991, *p* = 0.32) during the trial (Figure 2). However, this difference was not statistically significant. In contrast, a statistically significant decrease was found from t1 to t2 (β = 0.33 ± 0.13, t_174_ = 2.477, *p* = 0.015). No significant differences were found between the MGI measurements in the placebo group. As a result, there were significant differences between the placebo group and the treated group.

The LMM model for the values provided by the OH-15 questionnaire scores identified significant effects for variations in group and age but without any significant effects for measure, group × measure interaction, and sex (Table 3). There was a non-significant trend toward lower values in the OH-15 questionnaire, compared to t0 in the treated group, with a slight increase in the placebo group (Figure 2) that led to differences between the placebo group and the treated group at the various measurement points. In particular, the OH-15 questionnaire value was significantly lower than that of the placebo group at t3 (β = 5.3 ± 2.5, t_172_ = 2.086, *p* = 0.038). The OH-15 questionnaire value also decreased with increasing age (β = −0.11 ± 0.05, t_172_ = 2.297, *p* = 0.023) regardless of the measurement and treatment. 

The LMM model for the self-assessment VAS identified significant effects for the group and for the group × measure interaction regardless of sex and age (Table 3). In the treated group, the VAS decreased during the trial (Figure 2). The decrease was statistically significant from t0 to t1 (β = 0.73 ± 0.27, t_174_ = 2.725, *p* = 0.007) but not from t1 to t2 (β = 0.43 ± 0.27, t_174_ = 1.610, *p* = 0.11) nor from t2 to t3 (β = 0.47 ± 0.27, t_174_ = 1.734, *p* = 0.085). In contrast (Figure 2), in the placebo group, the VAS value increased significantly from t0 to t1 (β = 0.70 ± 0.27, t_174_ = 2.601, *p* = 0.010) and from t2 to t3 (β = 0.80 ± 0.27, t_174_ = 2.928, *p* = 0.0034) but not from t1 to t2 (β = 0.47 ± 0.27, t_174_ = 1.734, *p* = 0.085). Thus, the VAS index did not differ between groups at t0 (β = 0.44 ± 0.51, t_88_ = 0.872, *p* = 0.39) and t1 (β = 0.99 ± 0.51, t_88_ = 1.124, *p* = 0.052), while it was significantly lower than that of the placebo group at both the t2 (β = 1.89 ± 0.51, t_88_ = 3.746, *p* < 0.001) and the t3 (β = 3.16 ± 0.51, t_88_ = 6.253, *p* < 0.001) measurements.

Significant effects for the group and for the group x measure interaction, in the absence of effects due to sex and age, were found for the LMM model for the CGI-S questionnaire (Table 3). In the treated subjects’ group (Figure 2), the CGI-S questionnaire value decreased significantly both between t0 and t1 (β = 0.33 ± 0.16, t_174_ = 2.080, *p* = 0.040) and between t1 and t2 (β = 0.43 ± 0.16, t_174_ = 2.704, *p* = 0.075), while it was unchanged between t2 and t3 (β = 0.04 ± 0.16, t_174_ = 0.283, *p* = 0.78). In the placebo group, the CGI-S value significantly increased between t1 and t2 (β = 0.33 ± 0.16, t_174_ = 2.080, *p* = 0.039) and between t2 and t3 (β = 0.32 ± 0.16, t_174_ = 2.079, *p* = 0.039). As a result, the CGI-S index did not differ between groups at t0 (β = 0.19 ± 0.32, t_83_ = 0.593, *p* = 0.55) and t1 (β = 0.05 ± 0.32, t_83_ = 0.173, *p* = 0.86), while the scores were significantly lower than those of the placebo group at both t2 (β = 0.71 ± 0.32, t_83_ = 2.238, *p* = 0.029) and t3 measurements. (β = 1.09 ± 0.32, t_83_ = 3.419, *p* < 0.001).

Finally, during the three months of treatment, no subjects reported adverse reactions.

Related to the administration of the chewing gum, this supports the notion that this food supplement could be considered well tolerated.

## 4. Discussion

Gingivitis is an inflammatory condition of the gums and their tissue, mostly caused by bacterial infection [22]. Of all periodontal diseases, gingivitis is considered the most common. There are various forms of gingivitis based on their clinical appearance, duration of infection, severity, and etiology. The chronic form of gingivitis caused by plaque is the most frequent [23]. Clinically, swelling, redness, tenderness, shiny surface, and bleeding on probing are the hallmarks of gingivitis. However, gingivitis seldom generates spontaneous bleeding and is commonly painless, so many people do not recognize the incidence of the disease.

The potential beneficial activity of hydroalcoholic extracts obtained from the aerial part of *S. lateriflora* and *C. incanus* [4] was put to a clinical trial based on promising in vitro findings. These two components are used as food supplement ingredients and, in this study, were conveyed to the oral cavity in the form of a chewing gum, aiming to improve symptoms associated with gingivitis and stop its progression to periodontitis and in turn enhance the quality of life of the individual. Pertinent variables were assessed by means of validated questionnaires.

Taken together, following a 3-month use of the chewing gum, the results indicate a clinically relevant and statistically significant improvement in the gingival status of the subjects. The *QGBI* questionnaire quantitatively scores gingival bleeds following brushing and slight pressure on the gums. The following scale is employed: (0) no bleeding on brushing; absence of blood-stained bristles; (1) mild bleeding on brushing; presence of blood spots on the tips of the bristles; (2) moderate bleeding on brushing; about half the length of the bristles, from the tip down, stained with blood; (3) Severe bleeding on brushing; full length of all bristles. The LMM model for the QGBI index identified a significant sex- and age-independent, statistically significant decrease in gum bleeding, showing that for the treated group, the food supplement induced a statistically significant transition from a state of moderate bleeding on brushing (about half the length of the bristles, from the tip down) to a state of slight bleeding when brushing (presence of blood stains on the tips of the bristles). This clinically relevant improvement did not occur in the placebo group. The doctor’s visual assessment of the degree of inflammation of the gum tissue were evaluated according to the following scale: (0) normal gingiva; (1) mild inflammation—slight discoloration and slight edema; (2) moderate inflammation—redness, oedema, and glazing; (3) severe inflammation—marked redness and oedema, and ulceration with a tendency toward spontaneous bleeding. At the various measurement points, there was a statistically significant decrease in gum inflammation of the recruited subjects, consistent with the possibility of a food supplement-related improvement in the healthy status of gums, from moderate (characterized by redness, edema, and glazing) to a mild inflammation state (characterized by slight discoloration and slight edema) in the treated group. This agrees with the decreased discomfort reported by those treated with the dietary supplement containing *S. lateriflora* and *C. incanus* extracts, as judged via the *OH-15* self-assessment questionnaire, which was used to evaluate discomfort in the oral cavity in the 7 days prior to completion of the study. The *VAS*, which ranges from one (no pain) to ten (the highest level of perceived pain), was used to quantify the subject’s evaluation of the pain perceived at the level of the gum. A statistically significant reduction in gum pain was experienced by the recruited subjects following treatment with the chewing gum enriched with the *S. lateriflora* and *C. incanus* extracts. This improvement in symptoms was maintained over the following two months. On the other hand, in keeping with the fact that gingivitis tends to worsen over time if not properly treated, the worsening of pain was documented in subjects receiving the placebo. The *CGI-S* questionnaire, commonly used for a physician’s assessment of the general condition of gingivitis, employs the following scale: (1) normal, not at all sick; (2) at the limits of illness; (3) mildly ill; (4) moderately ill; (5) sick; (6) seriously ill; and (7) among those with the most severe degree of disease. Compared to those that received the placebo, the subjects that received the chewing gums enriched with *S. lateriflora* and *C. incanus* extracts for three months showed an improvement in their gingival status from “mildly ill” to a “borderline disease” according to the definitions used in the questionnaire. This argues for a statistically significant and clinically relevant improvement.

The rationale for the use of *S. lateriflora* and *C. incanus* was based on promising results in preclinical studies [4] as well as their common use in the preparation of food supplements. In particular, *S. lateriflora* extracts are known to combat gingivitis. On the other hand, attempts to target the main risk factors for periodontal disease typically focus on five aspects: antibacterial effect, inhibition of dental plaque formation, protective effects on periodontal tissues, regulatory effects on pro-inflammatory mediators and matrix metalloproteinases (MMP), and regulatory effects on the innate immune response [8]. Where the inhibition of plaque formation is concerned, certain bacteria present in the oral microbiota play a critical role in the early phases and in the progression of periodontal disease. Pathogenic bacteria activate host immunological cells, which produce various mediators and effectors of tissue breakdown [24]. Therefore, antibiofilm and antibacterial therapies are the treatment of choice in the control and treatment of periodontal disease. Some skullcap species have a potent antibacterial effect on oral pathogens [25]. Regarding the protective effect on periodontal tissues and regulatory effect on the gene expression of osteogenic markers, the goal of periodontal therapy is to control inflammation in the damaged tissue and promote tissue regeneration. Cells involved in this process are human periodontal ligament cells (hPDLCs) that differentiate into cementoblasts or osteoblasts. In vitro studies show that, by regulating the expression of osteogenic markers, including runt-related transcription factor 2 (RUNX2), bone morphogenetic protein 2 (BMP2), Osterix (OSX), osteocalcin (OCN), and the Wnt/β-catenin pathway, baicalin and baicalein are involved in the early and late stages of the osteogenic differentiation of hPDLCs. Indeed, baicalin and baicalein are components of the hydroalcoholic extract of *S. lateriflora* [4] used as an ingredient of the chewing gum tested in this clinical trial, leading to an improvement in periodontal tissue morphology, a significant increase in alveolar bone mineral density, and tissue repair and regeneration [8,26,27]. Regarding the effect on pro-inflammatory mediators and matrix MMP, inflammation of the periodontal pocket is a hallmark of periodontitis. The release of various inflammatory cytokines resulting from the host’s immune response has been reported to worsen gingivitis and alveolar bone resorption. Cytokines are involved in this event: IL-1β, which initiates the inflammatory response in infections; IL-6, which induces osteoclastogenesis and alveolar bone resorption in response to periodontal pathogens; and IL-8, which plays a key role in neutrophil migration into the periodontium. In addition, TNF-α stimulates the expression of matrix metalloproteinase, which in turn leads to collagen degradation in gum tissues [28]. In animal models, baicalin and baicalein, the bioactive compounds present in *S. lateriflora* extract, inhibit most cytokines involved in periodontitis [29]. Finally, considering the effect of the innate immune response, and the fact *Porphyromonas gingivalis* is one of the most important periodontal pathogens, the immune response against *P. gingivalis* plays a critical role in periodontal disease. The humoral immune response against this pathogen has been extensively evaluated in volunteers with chronic periodontitis. Compared to those without, individuals with chronic periodontitis have significantly higher IgG levels, along with an increase in the specific IgG2 response [30]. In addition, periodontitis in experimental animal models was found to cause a Th1-type immune response. Studies have also shown that baicalin present in skullcap extract maintains alveolar bone homeostasis in chronic periodontitis by means of exerting a positive effect on the mechanisms of innate immune responses, which also involve the action of Th1 and Th2 immune cells [30,31].

As far as *C. incanus* is concerned, it is a hybrid obtained from the cross between the two species *Cistus albidus* L. and *Cistus crispus* L. [32] and is very common in the Mediterranean basin. This plant is rich in essential oils, containing diterpenes and multiple polyphenolic compounds that protect the plant from environmental stressors, both biotic and abiotic, and make it resistant to extreme environmental conditions (nitrogen deficiency, high temperatures, etc.). Several species *of Cistus*, including *C. incanus*, are commonly used in traditional medicine [33]. Apigenin, kaempferide, and kaempferol acylated glycosides (cis- and trans-tiliroside and their conjugates with p-coumaric acid) represent a significant fraction of compounds present in leaves, stems, and flowers [34] and confer antipathogenic activity. Due to its remarkable antibacterial, viral, and antifungal activity, *C. incanus* is a valid choice for the treatment and prevention of periodontal disease. Indeed, the adjuvant action of the phytocomplex, especially the considerable polyphenolic fraction, exerts a significant action on pathogens of the oral cavity. Specifically, there is documented inhibitory activity of plant extracts from *C. incanus* against bacterial growth, their colonization and adhesion in the oral cavity, and the decrease in bacterial peroxidase [35], for instance, results on *Staphylococcus aureus*, *Escherichia coli*, and *Streptococcus mutans* [36]. The use of a product containing *C. incanus*, used for rinsing the oral cavity, confirmed its antibacterial properties and supported the efficacy and the impact of this extract [37].

There are, however, certain limitations with the current study. The discrepancy between the treatment and the placebo groups with respect to age is certainly a limitation of the present investigation. However, it did not significantly affect the efficacy evaluation of the treatment. This further supports the decision to use the simple randomization model rather than a block randomization as insufficient blinding of the trial is a common disadvantage for small block size sequences such as the present one. Another possible limitation to the present study is the lack of control of the food intake and the diet composition for each participant. Nevertheless, the use of a food diary was not adopted because the study aims to assess the efficacy of this food supplement in the general population, which is characterized by heterogeneity in diet composition. Another possible source of heterogeneity is the slightly different taste of the chewing gum used for treatment group and placebo group. To try to avoid this source of heterogeneity, the placebo and food supplement production process included sensory testing to bring the taste of the food supplement as close as possible to the taste of the placebo, using food flavours. Moreover, it is also important to highlight that the parallel-group clinical trial was conducted in a double-blind setting. Therefore, neither the subject nor the investigator was aware of the treatment administered to the participants. Therefore, although there had been slight differences in taste between the food supplement and the placebo, these differences did not affect the study results. For instance, studies in patients with congenital bleedings argue for gingival bleeding data as a reliable index regardless of the triggering conditions [38,39]. The manner in which the observations were carried out in the present study may have biased the evaluations and influenced the results. However, despite their inherent subjectivity, validated questionnaires have been employed for all the evaluations carried out in this clinical double-blinded trial, and all agreed with the bleeding data. This suggests that the parameters gathered across the three-month observation period (by the volunteers and the physician) in this study support and extend the results of the bleeding indices, lending credence toward the possibility that the present data identify new important directions to be pursued by studies in this field.

Perspectives in the area also emerge from the present data collection. Chronic gum inflammation has been associated with systemic diseases (e.g., respiratory, coronary, diabetes, stroke, rheumatoid arthritis). Future research will focus on the bacteria responsible for periodontitis which are able to enter the bloodstream through gum tissue, eventually affecting the heart, lungs, and other parts of the body. [32] The use of saliva samples is now widely accepted for determining the oral microbiota. At different time intervals during the study, the participants were provided with sterile saliva collection tubes, along with collection instructions, for identifying the microbial species and strains present (oral microbiota analysis). Future next-generation sequency (NGS) determinations will evaluate whether the use of *S. lateriflora* and the *C. incanus* extracts included as ingredients of the food supplement in the form of a chewing gum will be also able to affect the oral microbiota.

## 5. Conclusions 

In conclusion, the results indicate a clinically relevant and statistically significant improvement in the gingival status of the subjects, following 3-month use of this food supplement, which was well tolerated and may provide a suitable treatment to reduce the symptoms of gingivitis and prevent the progression of gingivitis to periodontitis.

## Figures and Tables

**Table 1:** Characteristics of subjects enrolled in the study.

Characteristics of Enrolled Subjects	Group 1, Placebo (*n* = 30)	Group 2, Supplement (*n* = 30)
**Ethnicity**		
Caucasian	30	30
**Gender**		
Males (*n*)	13	17
Females (*n*)	17	13
**Age (years)**		
Males	35.8 ± 12.0	51.5 ± 12.1
Females	37.5 ± 11.8	47.3 ± 16.5
Median age (years, range)	38.5 ± 14.1 (20–68)	48.5 ± 14.4 (22–68)

**Table 2:** Values of the five outcomes evaluated (mean ± standard deviation, minimum–maximum) at the four evaluation timepoints in the placebo and in the treatment group.

Variable	Placebo Group (*n* = 30)				Treatment Group (*n* = 30)			
	t0	t1	t2	t3	t0	t1	t2	t3
QGBI	1.9 ± 0.8	1.7 ± 0.8	1.7 ± 0.7	2.0 ± 0.8	2.3 ± 0.9	1.9 ± 0.7	1.7 ± 0.7	1.1 ± 0.7
	0–3	0–3	0–3	0–3	0–3	0–3	0–3	0–3
MGI	2.0 ± 0.7	1.9 ± 0.7	1.9 ± 0.7	2.0 ± 0.7	1.8 ± 1.0	1.6 ± 0.9	1.2 ± 0.7	1.1 ± 0.7
	1–3	1–3	1–3	1–3	0–3	0–3	0–2	0–3
0H-15	36.1 ± 10.6	36.8 ± 10.1	36.8 ± 10.1	38.5 ± 10.2	34.8 ± 12.2	33.4 ± 11.6	31.8 ± 10.9	30.5 ± 11.3
	16–50	18–51	17–49	18–51	17–54	17–52	16–50	16–53
VAS	4.1 ± 2.0	4.8 ± 1.8	5.2 ± 1.9	6.0 ± 2.0	4.1 ± 2.3	3.4 ± 1.9	3.0 ± 1.7	2.5 ± 1.7
	1–8	1–8	2–8	2–8	0–8	0–7	0–7	0–7
CGI-S	3.2 ± 1.3	3.0 ± 0.9	3.3 ± 1.0	3.7 ± 1.2	3.3 ± 1.4	3.0 ± 1.2	2.5 ± 1.1	2.4 ± 1.1
	1–6	1–5	1–5	1–6	1–6	1–5	1–6	1–5

**Table 3:** LMM model results for variables associated with the primary outcomes.

Model	GDL NUM	GDL ON	F	*p* Value
QGBI				
Measure	3	174	10.93	<0.001
Group	1	56	0.179	0.67
Sex	1	56	0.222	0.64
Age	1	56	0.156	0.69
Size x Group	3	174	18.48	<0.001
MGI				
Measure	3	174	4.663	0.0036
Group	1	56	7.889	0.0068
Sex	1	56	0.120	0.73
Age	1	56	0.970	0.33
Size x Group	3	174	6.054	<0.001
OH-15 (relative)				
Measure	2	172	0.113	0.89
Group	1	172	5.412	0.021
Sex	1	172	3.876	0.051
Age	1	172	5.278	0.023
Size x Group	2	172	1.063	0.35

**Table 4:** LMM model results for variables associated with the secondary outcomes.

Model	GDL NUM	GDL ON	F	*p*
VAS				
Measure	3	174	0.413	0.74
Group	1	56	9.769	0.0028
Sex	1	56	2.957	0.091
Age	1	56	3.668	0.061
Size x Group	3	174	31.72	<0.001
CGI-S				
Measure	3	174	3.028	0.031
Group	1	56	1.854	0.18
Sex	1	56	0.107	0.74
Age	1	56	0.692	0.41
Size x Group	3	174	14.49	<0.001

## Data Availability

The original contributions presented in the study are included in the article, further inquiries can be directed to the corresponding authors.
